# Survival differences between patients with de novo and relapsed/progressed advanced non-small cell lung cancer without epidermal growth factor receptor mutations or anaplastic lymphoma kinase rearrangements

**DOI:** 10.1186/s12885-023-10950-y

**Published:** 2023-05-29

**Authors:** Byeong-Chan Oh, Ae-Ryeo Cho, Jin Hyun Nam, So-Young Yang, Min Ji Kim, Sun-Hong Kwon, Eui-Kyung Lee

**Affiliations:** 1 School of Pharmacy, Sungkyunkwan University, 2066 Seobu-ro, Jangan-gu, Suwon, Gyeonggi- do Republic of Korea; 2 Division of Big Data Science, Korea University Sejong Campus, Sejong-si, Republic of Korea; 3 Amgen Korea Limited, Seoul, Republic of Korea

**Keywords:** Non-small cell lung cancer, Overall survival, Treatment pattern, De novo, Recurrence, Real-world data

## Abstract

**Background:**

We aimed to examine whether patients with de novo and relapsed/progressed stage IIIB–IV non-small cell lung cancer (NSCLC) without epidermal growth factor receptor (*EGFR*) or anaplastic lymphoma kinase (*ALK*) mutations have different prognoses.

**Methods:**

This retrospective study analyzed the Health Insurance Review and Assessment claims data in South Korea from 2013 to 2020. Patients with stage IIIB–IV NSCLC without *EGFR* or *ALK* mutations who received first-line palliative therapy between 2015 and 2019 were identified. Overall survival (OS), time to first subsequent therapy (TFST), and time to second subsequent therapy (TSST) were estimated using the Kaplan–Meier method. Multivariate Cox regression analysis was used to reveal the impact of de novo versus relapsed/progressed disease on OS. Treatment patterns, including treatment sequence, top five most frequent regimens, and time to treatment discontinuation, were described in both groups.

**Results:**

Of 14,505 patients, 12,811 (88.3%) were de novo, and 1,694 (11.7%) were relapsed/progressed. The median OS in the de novo group was 11.0 versus 11.5 months in the relapsed/progressed group (*P* = 0.002). The ongoing treatment probability was higher in relapsed/progressed patients than in de novo patients from 6.4 months since the initiation of first-line treatment (*P* < 0.001). Median TSST was shorter in the de novo group than in the relapsed/progressed group (9.5 vs. 9.9 months, *P* < 0.001). In multivariate analysis, de novo disease was associated with shorter OS (hazard ratio 1.07; 95% confidence interval 1.01–1.14). The overall treatment patterns for de novo and relapsed/progressed patients were similar.

**Conclusions:**

De novo patients had poorer OS and TSST after the initiation of palliative therapy than relapsed/progressed patients. These findings suggest that the stage of the disease at the time of initial diagnosis should be considered in observational studies and clinical trials as a prognostic factor.

**Supplementary Information:**

The online version contains supplementary material available at 10.1186/s12885-023-10950-y.

## Background

Lung cancer remains the leading cause of cancer-related deaths, accounting for 18% (2.2 million) of new deaths annually, and ranks second in incidence worldwide (11.4%, 1.79 million) [1]. The majority of initial diagnoses of lung and bronchus cancers occur when cancer has spread to regional lymph nodes (22%) or has metastasized (55%) [2]. Non-small cell lung cancer (NSCLC), which constitutes 84% of lung cancer cases, has a poor prognosis [3]. The 5-year relative survival rates were 26% for patients with NSCLC and 8% for patients with metastatic NSCLC in the United States [4]. NSCLC is a molecularly heterogeneous disease subdivided into various molecular subtypes based on genetic alterations. As a result of advances in targeted therapies for various biomarkers, such as epidermal growth factor receptor (*EGFR*) and anaplastic lymphoma kinase (*ALK*), and immunotherapies targeting programmed death-ligand 1 (PD-L1), the landscape of advanced NSCLC treatment is changing [5]. Treatment options for patients with advanced NSCLC without *EGFR* or *ALK* mutations, such as platinum-based regimens, were limited to conventional chemotherapy before the emergence of immunotherapy, which has been reimbursed in South Korea since August 2017. Although the development of immunotherapies has improved survival [6], the prognosis for patients with advanced NSCLC remains poor. A recent chart review study in Japan reported a median overall survival (OS) of 21.1 months from the initiation of first-line therapy in patients with advanced NSCLC without actionable mutations [7].

While some studies have focused on patients with *EGFR* mutations [8, 9], it remains unclear whether patients with stage IIIB–IV NSCLC without *EGFR* or *ALK* mutations have a different prognosis according to the disease stage at the time of the initial diagnosis. De novo patients who are directly diagnosed with stage IIIB–IV NSCLC have shorter OS and progression-free survival (PFS) than relapsed/progressed patients [9–12]. Most of these studies used single-center data and were conducted in the pre-immunotherapy era, and no studies were conducted on patients without *EGFR* or *ALK* mutations. In addition, a study published in 2021 reported that OS and PFS were not affected by the de novo versus secondary metastatic setting in patients with *EGFR*-mutated NSCLC [8]. In breast cancer, the association between prognosis and disease stage at the time of initial diagnosis has been reported in the opposite direction, with de novo patients having better survival [13, 14]. This suggests that the impact of de novo versus relapsed/progressed disease on survival may not be uniform across tumor sites and biomarker status. In addition, there is a need to understand the treatment patterns that may impact the survival of patients with de novo and relapsed/progressed NSCLC. Although previous studies have described treatment patterns of patients with advanced NSCLC [15–18], limited data exist describing real-world treatment patterns in both groups of patients.

In this study, we aimed to determine whether there are survival differences between de novo and relapsed/progressed disease in patients with stage IIIB–IV NSCLC without *EGFR* or *ALK* mutations. Furthermore, we observed the treatment patterns of the first to third lines of therapy in both de novo and relapsed/progressed patients in real-world settings using a nationwide health insurance claims database.

## Methods

### Data source

This study utilized national claims data from the Health Insurance Review and Assessment Service (HIRA) database in South Korea. The database is a useful source of data for generating real-world evidence with high generalizability, as it covers approximately 98% of the total population of Korea [19]. It contains comprehensive information about demographic characteristics, including sex, age, insurance type, and healthcare services, such as medical procedures, prescriptions, and records of diagnoses according to the International Classification of Disease-10th revision (ICD-10) for approximately 50 million beneficiaries.

### Study design and population

This retrospective cohort study used claims data from January 1, 2013 to October 30, 2020 (study period). Our study population comprised patients with stage IIIB–IV NSCLC without *EGFR* or *ALK* mutations. To select eligible patients from the claims data, patients with more than one diagnosis of lung cancer according to the ICD-10 code (C34) were identified. Patients who had received regimens for only SCLC as first-line palliative therapy, which was a proxy to rule out SCLC patients, were excluded. We excluded patients who had used *EGFR* or *ALK* tyrosine kinase inhibitors during the study period as an alternative to select patients without *EGFR* or *ALK* mutations. We adapted an operational definition used in a previous study to identify patients with stage IIIB–IV NSCLC, as the HIRA database does not provide information on the clinical stage [20]. Patients satisfying at least one of the following criteria during the index period (January 1, 2015 to October 30, 2019) were considered stage IIIB–IV NSCLC who initiated first-line palliative therapy: patients who (1) had used cytotoxic chemotherapy at least 6 months after thoracic surgery, as chemotherapy within 6 months after thoracic surgery was considered adjuvant therapy as an operational definition; (2) had used cytotoxic chemotherapy for the first time with no history of thoracic surgery, or (3) had undergone immunotherapy. The date of the initiation of first-line palliative therapy for advanced NSCLC was defined as the index date. Only adult patients (age ≥ 18 years) were included; patients with other cancers 2 years prior to the index date were excluded. Patients with pre-existing stage IIIB–IV NSCLC who underwent treatment for stage IIIB–IV NSCLC in 2013–2014, except for regimens for adjuvant therapy, were excluded. Patients who underwent thoracic surgery in 2013–2014 and received first-line chemotherapy between 6 months after thoracic surgery and January 1, 2015, were excluded to prevent misclassification of the line of therapy.

Among the patients with stage IIIB–IV NSCLC without *EGFR* or *ALK* mutations, de novo and relapsed/progressed patients were classified based on the history of thoracic surgery or NSCLC treatments 2 years prior to the index date. De novo patients were identified with no history of thoracic surgery or NSCLC treatment, including all regimens for adjuvant and palliative therapies. Other patients were identified as relapsed/progressed. Patients in both groups were followed up until either death or the end of the study period (October 30, 2020). The study design is illustrated in S1 Fig., and the treatment lists used for patient selection are shown in S1 Table.

### Study measures

OS, time to first subsequent therapy or death (TFST), and time to second subsequent therapy or death (TSST) were used to evaluate the prognosis. OS was calculated from the date of the initiation of first-line therapy to the date of death. The date of death was extracted from the claims data as follows: (1) death indication as a result of treatment; (2) ICD-10 codes I46.1, R96, R98, or R99; or (3) if there was no inpatient or outpatient medical record for 6 months. The operational definition was validated in a previous study when using the definition, the true-positive rate was over 98%, and the false-positive rate was less than 2% in patients with lung cancer in South Korea [21]. TFST and TSST were included to reflect the duration of disease and symptom control, considering patient compliance and tolerance because it was not possible to assess the date of progression from the claims data [22]. TFST was defined as the time from the date of the initiation of first-line palliative therapy to the start date of second-line therapy or death. TSST was defined as the interval between the initiation of first-line palliative therapy and the start of third-line therapy or death. The subsequent line of therapy was defined as the initiation of a new drug regimen after the initial 28-day period. The discontinuation of some agents in the initial regimen was not considered a change in the line of therapy. The line of therapy did not change when there was an exchange of cisplatin or carboplatin or when the new drug regimen was maintenance therapy with pemetrexed.

We assessed the top five most frequent regimens in first- and second-line therapies. For the top five regimens, the number of patients and time to treatment discontinuation were observed as measures of treatment duration. The time to treatment discontinuation was defined as the time until treatment discontinuation or death. The date of treatment discontinuation was calculated by adding the length of the administration cycle to the date of the last prescription. The regimens included in the analysis were determined according to the HIRA reimbursement list in South Korea (S2 Table).

The patient characteristics identified on the index date included age, sex, Charlson comorbidity index (CCI), type of insurance, type of hospital, geographic region of the hospital, and the time point at the initiation of first-line therapy. CCI was computed over the 1-year pre-index period to assess patients’ baseline comorbidity status; lung cancer diagnosis was excluded from the CCI algorithm [23]. The time point at the initiation of first-line palliative therapy was classified based on the reimbursement of immunotherapy for second-line therapy; if first-line palliative therapy was initiated between 2018 and 2019, it was defined as the post-immunotherapy era and before 2018, it was defined as the pre-immunotherapy era. We designated the post-immunotherapy era as the time period in South Korea when the use of immunotherapeutic agents was believed to have commenced in earnest, aligning with the initiation of reimbursement for immunotherapies in August 2017.

### Statistical analysis

Patient characteristics of the study population are summarized descriptively. OS, TFST, and TSST were estimated using the Kaplan–Meier method, and patients who did not experience the event during the study period were censored at the dataset cut-off date. To focus on patients who survived beyond 1 year after the initiation of first-line therapy, we also counted survival from the 1-year landmark using the Kaplan–Meier method. Survival curves for de novo and relapsed/progressed patients were compared using log-rank tests. A multivariate Cox proportional hazards model was used to determine the impact of de novo versus relapsed/progressed disease in terms of OS and TFST while adjusting for measured confounders, such as age group, sex, CCI, insurance type, type of hospital, geographic region of hospital, and the time point at the initiation of first-line therapy. Treatment sequences for up to the first three lines of therapy are illustrated using a Sankey diagram. We explored the operational definition of adjuvant therapy through a sensitivity analysis to investigate its effect on OS. In the sensitivity analysis, cytotoxic chemotherapy used within 5 months was considered adjuvant therapy.

All analyses were conducted using SAS Enterprise Guide version 7.1 (SAS Institute, Inc., Cary, North Carolina, USA) and R (version 3.5.1) (The R Foundation for Statistical Computing, Vienna, Austria). The SAS Enterprise Guide software was used for data management and analyses, and R was used to create Sankey diagrams and Kaplan–Meier survival curves. A two-tailed value of *P* < 0.05 was considered statistically significant.

## Results

### Patient characteristics

In the HIRA database, 293,199 patients were diagnosed with lung cancer between January 2013 and October 2020. Of these, 28,673 patients with stage IIIB–IV NSCLC without *EGFR* or *ALK* mutations were identified. After excluding patients who had previously been in stage IIIB–IV and those with other cancers, a total of 14,505 patients were included in the study. Among them, 12,811 (88.3%) were de novo patients, and 1,694 (11.7%) were relapsed/progressed patients (S2 Fig.). The median follow-up duration was 11.1 months (interquartile range, 5.0–21.0 months) from the initiation of first-line palliative therapy.

Table 1 summarizes the patient characteristics stratified by de novo and relapsed/progressive diseases. The mean age at initiation of first-line palliative therapy was 67.6 years (standard deviation [SD], 9.2) for de novo patients and 66.7 years (SD, 8.5) for relapsed/progressed patients. Most patients in each group were male (87.1% and 87.0%) and covered by National Health Insurance (92.9% and 92.8%). The CCI score was ≥ 3 points in 35.8% and 37.1% of de novo and relapsed/progressed patients, respectively.

**Table 1:** Baseline characteristics of study population, n (%)

		Total (n = 14,505)	De novo patients (n = 12,811)	Relapsed/progressed patients (n = 1,694)
**Age group at initiation of first-line therapy, years**				
	< 60	2,682 (18.5)	2,354 (18.4)	328 (19.4)
	60–69	5,269 (36.3)	4,578 (35.7)	691 (40.8)
	70–79	5,499 (37.9)	4,894 (38.2)	605 (35.7)
	≥ 80	1,055 (7.3)	985 (7.7)	70 (4.1)
**Sex**				
	Male	12,633 (87.1)	11,160 (87.1)	1,473 (87.0)
	Female	1,872 (12.9)	1,651 (12.9)	221 (13.1)
**CCI**				
	< 3	9,290 (64.1)	8,225 (64.2)	1,065 (62.9)
	≥ 3	5,215 (36.0)	4,586 (35.8)	629 (37.1)
**Insurance type**				
	National health insurance	13,479 (92.9)	11,907 (92.9)	1,572 (92.8)
	Medical aid or veterans	1,026 (7.1)	904 (7.1)	122 (7.2)
**Type of hospital at initiation of first-line therapy**				
	Tertiary hospital	10,512 (72.5)	9,251 (72.2)	1,261 (74.4)
	Others	3,993 (27.5)	3,560 (27.8)	433 (25.6)
**Geographic region of hospital**				
	Capital area	9,408 (64.9)	8,220 (64.2)	1,188 (70.1)
	Metropolitans	2,753 (19.0)	2,477 (19.3)	276 (16.3)
	Rural	2,344 (16.2)	2,114 (16.5)	230 (13.6)
**Time point at initiation of first-line therapy**				
	Pre-immunotherapy era	8,117 (56.0)	7,194 (56.2)	923 (54.5)
	Post-immunotherapy era	6,388 (44.0)	5,617 (43.9)	771 (45.5)

CCI, Charlson comorbidity index

All percentages may not be a total of 100% due to rounding.

### Clinical outcomes

**Fig. 1:**
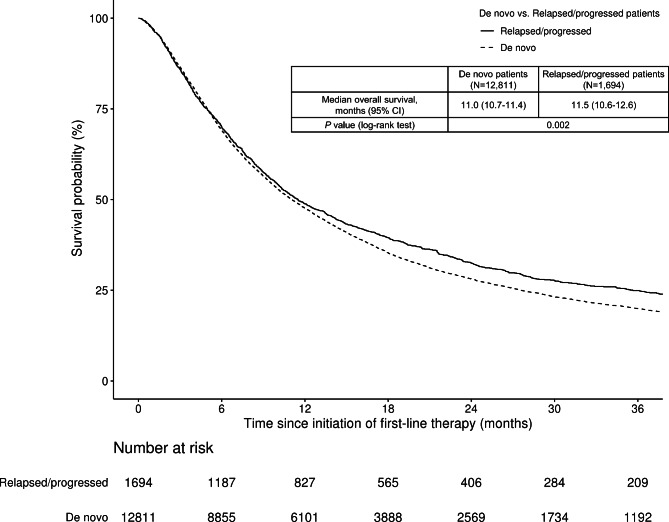
Overall survival in de novo and relapsed/progressed advanced non-small cell lung cancer CI, confidence interval

**Fig. 2:**
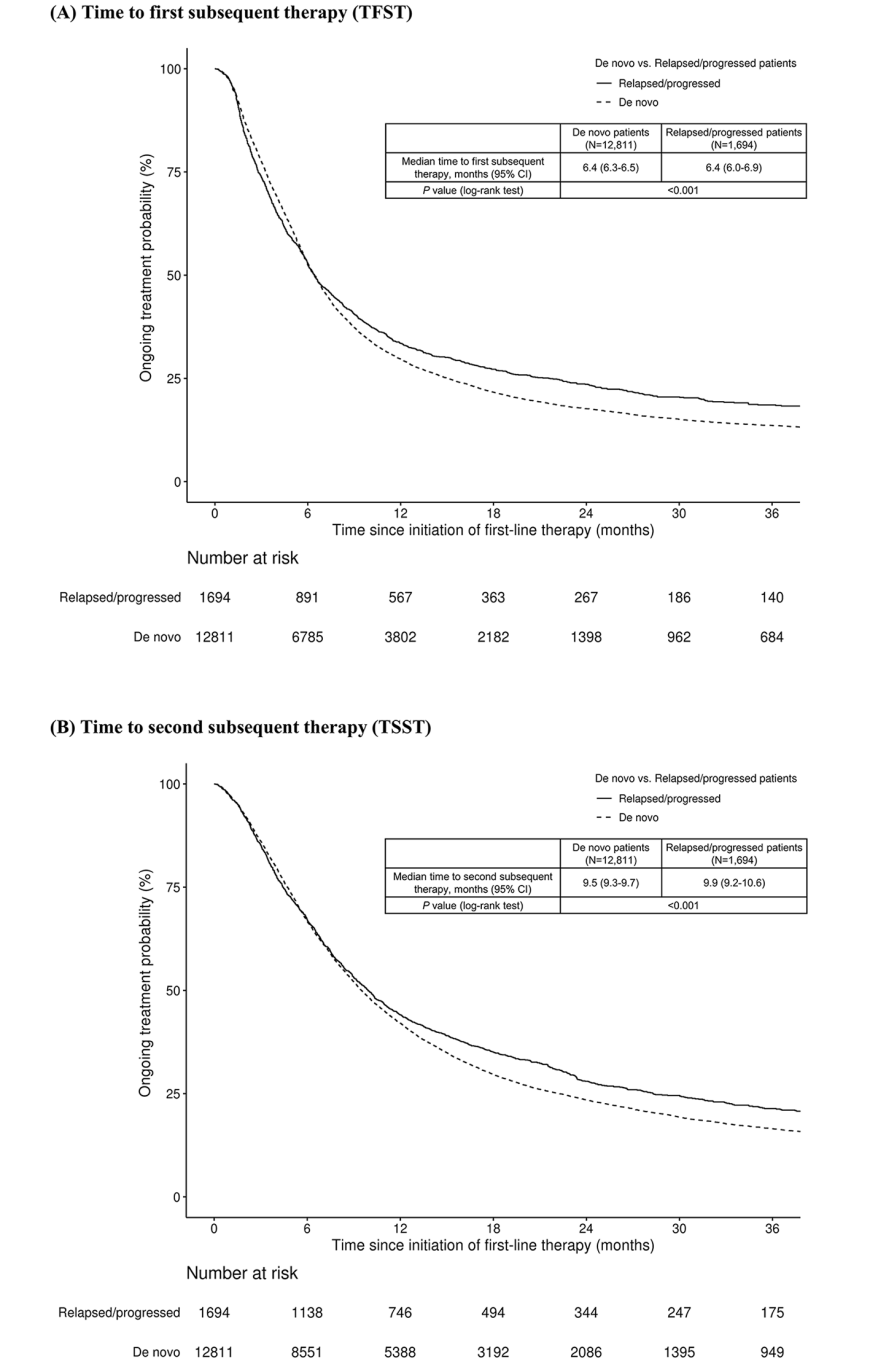
Time to (**A**) first and (**B**) second subsequent therapy or death in de novo and relapsed/progressed advanced non-small cell lung cancer CI, confidence interval

By data cutoff, 76.5% (9,804/12,811) of the de novo and 73.1% (1,239/1,694) of the relapsed/progressed patients had died. There was a statistically significant difference (*P* = 0.002) in OS from the initiation of first-line palliative therapy, with de novo patients presenting a median OS of 11.0 months (95% confidence interval [95% CI], 10.7–11.4 months) compared with 11.5 months (95% CI, 10.6–12.6 months) for relapsed/progressed patients (Fig. 1). The difference remained significant after changing the operational definition of adjuvant therapy to consider cytotoxic chemotherapy used within 5 months as adjuvant therapy in the sensitivity analysis (S3 Fig.). Kaplan-Meier survival curves were generated for patients who survived beyond 1 year after the initiation of first-line therapy, with survival counted from the 1-year landmark. The analysis of the survival data showed that the curves for de novo and relapsed/progressed patients diverged over time, with a significant difference observed (S4 Fig.). Stratification of Kaplan–Meier curves for OS based on immunotherapy use was also presented, which still revealed a significant difference (S5 Fig., S4 Table). Median TFST was 6.4 months (95% CI, 6.3–6.5 months) in de novo patients and 6.4 months (95% CI, 6.0–6.9 months) in relapsed/progressed patients (*P* < 0.001) (Fig. 2a). Median TSST in de novo patients was 9.5 months (95% CI, 9.3–9.7 months) versus 9.9 months (95% CI, 9.2–10.6 months) in relapsed/progressed patients (*P* < 0.001) (Fig. 2b).

**Fig. 3:**
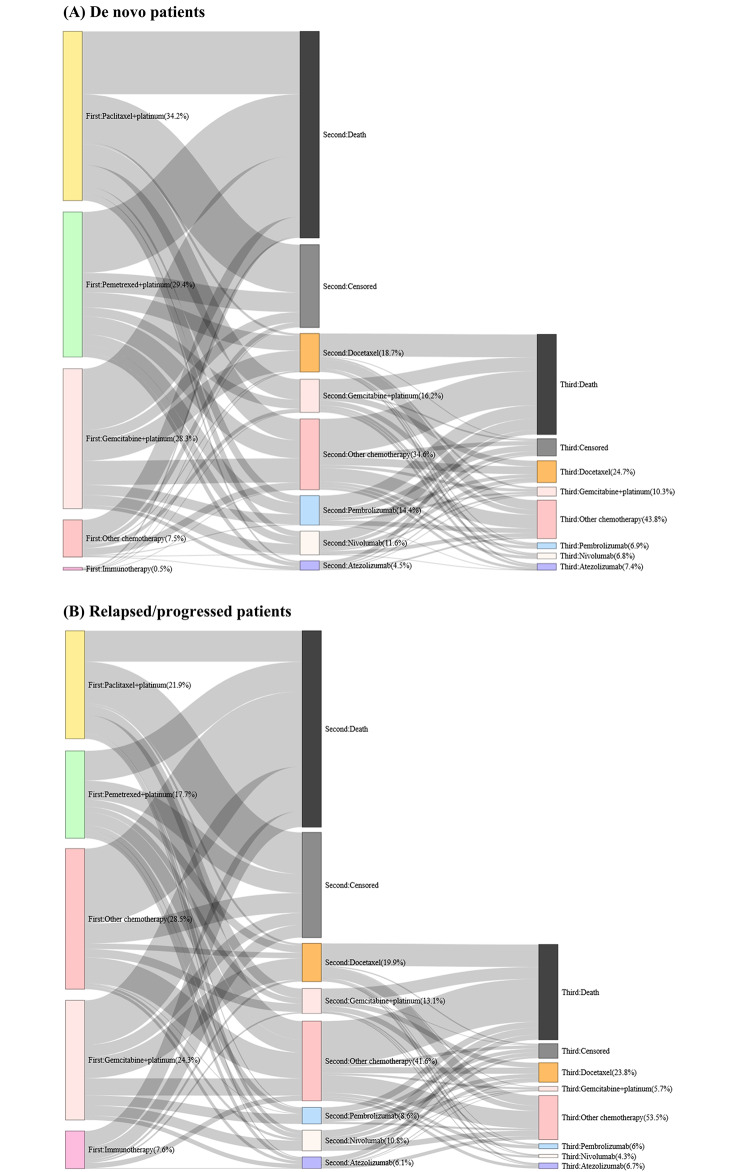
Sankey diagram of treatment patterns (**A**) De novo patients; (**B**) Relapsed/ progressed patients

Table 2 presents the results of the multivariate Cox regression analysis with hazard ratios > 1.0 meaning an increased risk of death. According to the analysis, de novo disease was associated with shorter survival than relapsed/progressed disease after adjusting for confounders (hazard ratio [HR], 1.07; 95% CI, 1.01–1.14). Other known prognostic factors, including older age, male sex, and a higher comorbidity index, were also correlated with worse survival. Insurance type, type of hospital, and geographic region of the hospital were associated with prognosis in de novo patients but not in relapsed/progressed patients. De novo patients who initiated first-line palliative therapy in the post-immunotherapy era showed a better prognosis than those who initiated therapy in the pre-immunotherapy era (HR, 0.83; 95% CI, 0.79–0.86). The results of multivariate Cox regression analysis for TFST are shown in S3 Table.

**Table 2:** Hazard ratio for overall survival

		Hazard ratio (95% CI)		
Variable		Total (n = 14,505)	De novo patients (n = 12,811)	Relapsed/progressed patients (n = 1,694)
De novo vs. relapsed/progressed				
	Relapsed/progressed	reference	-	-
	De novo	1.07 (1.01–1.14)	-	-
Age group, years				
	< 60	reference	reference	reference
	60–69	1.15 (1.09–1.22)	1.17 (1.10–1.24)	1.04 (0.89–1.22)
	70–79	1.41 (1.33–1.49)	1.44 (1.36–1.53)	1.20 (1.03–1.41)
	≥ 80	1.72 (1.59–1.87)	1.73 (1.59–1.88)	1.85 (1.39–2.47)
Sex				
	Female	reference	reference	reference
	Male	1.37 (1.29–1.45)	1.35 (1.27–1.44)	1.54 (1.28–1.85)
CCI				
	< 3	reference	reference	reference
	≥ 3	1.08 (1.03–1.12)	1.07 (1.02–1.11)	1.14 (1.01–1.28)
Insurance type				
	National health insurance	reference	reference	reference
	Medical aid or veterans	1.16 (1.08–1.24)	1.15 (1.06–1.24)	1.24 (1.00–1.54)
Type of hospital at initiation of first-line therapy				
	Tertiary hospital	reference	reference	reference
	Others	1.06 (1.02–1.11)	1.07 (1.02–1.11)	1.02 (0.89–1.16)
Geographic region of hospital				
	Capital area	reference	reference	reference
	Metropolitans	1.09 (1.04–1.15)	1.11 (1.06–1.17)	0.96 (0.82–1.13)
	Rural	1.09 (1.03–1.14)	1.09 (1.03–1.15)	1.08 (0.91–1.27)
Time point at initiation of first-line therapy				
	Pre-immunotherapy era	reference	reference	reference
	Post-immunotherapy era	0.84 (0.80–0.87)	0.83 (0.79–0.86)	0.90 (0.80–1.02)

CCI, Charlson comorbidity index; CI, confidence interval

### Treatment patterns

The proportion of patients who died after initiating first-line palliative therapy without any subsequent therapy was 41.8% and 39.8% in the de novo and relapsed/progressed groups, respectively. During the first-line palliative therapy, 16.7% and 21.3% of patients were censored at the end of the study period in de novo and relapsed/progressed patients, respectively. Table 3 presents the top five regimen compositions for first- and second-line therapy, and Fig. 3 illustrates the treatment sequences by regimen. Platinum-based chemotherapy was primarily used as first-line therapy in both groups of patients. The most frequently used regimens were paclitaxel + platinum, pemetrexed + platinum, and gemcitabine + platinum. These three regimens were predominant in both de novo (34.2%, 29.4%, and 28.3%) and relapsed/progressed (21.9%, 17.7%, and 24.3%) patients. Relapsed/progressed patients used a more varied therapeutic approach for first-line therapy than de novo patients.

**Table 3:** Top five regimens and their time to discontinuation

	Number of patients (%)			Time to treatment discontinuation (months), mean (SD)		
	Total	De novo patients	Relapsed/progressed patients	Total	De novo patients	Relapsed/progressed patients
First-line regimen	14,505 (100.0)	12,811 (100.0)	1,694 (100.0)	2.9 (2.7)	2.8 (2.5)	3.0 (4.0)
Paclitaxel + platinum	4,758 (32.8)	4,387 (34.2)	371 (21.9)	2.8 (2.9)	2.8 (2.9)	2.5 (2.8)
Pemetrexed + platinum	4,063 (28.0)	3,763 (29.4)	300 (17.7)	2.7 (1.3)	2.7 (1.3)	2.6 (1.1)
Gemcitabine + platinum	4,042 (27.9)	3,631 (28.3)	411 (24.3)	2.9 (2.7)	2.9 (2.4)	3.1 (4.3)
Gemcitabine	430 (3.0)	337 (2.6)	93 (5.5)	2.8 (4.8)	2.8 (5.2)	2.6 (3.0)
Docetaxel + platinum	329 (2.3)	295 (2.3)	34 (2.0)	3.1 (2.1)	3.2 (2.1)	2.2 (1.7)
Others	883 (6.1)	398 (3.1)	485 (28.6)	3.6 (4.9)	3.4 (4.1)	3.7 (5.4)
Second-line regimen	5.973 (100.0)	5,314 (100.0)	659 (100.0)	3.8 (4.9)	3.7 (4.9)	3.8 (5.1)
Docetaxel	1,127 (18.9)	996 (18.7)	131 (19.9)	2.4 (2.3)	2.4 (2.3)	2.3 (1.9)
Gemcitabine + platinum	946 (15.8)	860 (16.2)	86 (13.1)	2.6 (2.3)	2.6 (2.4)	2.5 (1.9)
Pembrolizumab	822 (13.8)	765 (14.4)	57 (8.7)	7.0 (7.6)	6.9 (7.5)	8.7 (8.6)
Nivolumab	688 (11.5)	617 (11.6)	71 (10.8)	6.0 (7.0)	5.9 (6.9)	6.6 (8.1)
Paclitaxel + platinum	404 (6.8)	373 (7.0)	31 (4.7)	2.7 (3.9)	2.7 (3.8)	3.3 (5.3)
Others	1,986 (33.3)	1,703 (32.1)	283 (42.9)	3.2 (3.9)	3.1 (3.8)	3.3 (3.9)

SD, standard deviation

All percentages may not add to a total of 100% because of rounding.

Of the 12,811 and 1,694 patients who received first-line therapy in de novo and relapsed/progressed patients, 5,314 (41.5%) and 659 (38.9%) initiated second-line therapy, respectively. The most common second-line treatment regimen in both groups was docetaxel, administered to 18.7% and 19.9% of de novo and relapsed/progressed patients, respectively (Table 3; Fig. 3). Gemcitabine + platinum was the second-most preferred second-line therapy, administered to 16.2% and 13.1% of de novo and relapsed/progressed patients, respectively. The time to treatment discontinuation of the top five regimens for first- and second-line therapies is presented in Table 3 and S5 Table. The difference in the mean time to treatment discontinuation for first-line (2.8 vs. 3.0 months) and second-line therapies (3.7 vs. 3.8 months) between de novo and relapsed/progressed patients was less than 5 days.

## Discussion

In this population-based study of patients with stage IIIB–IV NSCLC without *EGFR* or *ALK* mutations, we found that de novo patients had worse clinical outcomes in terms of OS and TSST than relapsed/progressed patients. This difference remained significant, even after controlling for age, sex, and other factors. The overall treatment patterns for both de novo and relapsed/progressed patients were similar, except that relapsed/progressed patients used more varied therapeutic approaches as first-line therapy.

Although this study targeted patients with NSCLC without *EGFR* or *ALK* mutations and included the post-immunotherapy era, our findings were consistent with previous studies targeting patients with different biomarker status or studies conducted in the pre-immunotherapy era [9–12]. Our results showed that de novo patients had shorter median OS than relapsed/progressed patients (11.0 vs. 11.5 months; HR 1.07) from the initiation of first-line palliative therapy. According to previous studies in Canada that identified patients with metastatic NSCLC in the pre-immunotherapy era regardless of biomarker status, de novo presentation of metastatic NSCLC had HRs ranging from 1.2 to 1.4 in terms of OS than relapsed/progressed presentation [10, 11]. These studies reported the median OS from the date of diagnosis of metastatic disease ranged from 3.7 to 4.7 months for de novo patients and 6.9–8.9 months for relapsed/progressed patients. Similar results were reported in a study using single-center data from the United States, in which the median OS from the date of diagnosis of metastatic disease in patients with KRAS-mutant lung adenocarcinomas was 13 months in de novo patients and 18 months in recurrent patients (HR, 1.41) [9]. These results are in line with our findings that more patients in the de novo group died during first-line therapy than in the relapsed/progressed group (41.8% vs. 39.8%). In addition, fewer patients in the de novo group reached the end of the study period without initiation of second-line therapy or death (16.7% vs. 21.3%).

Other than the OS, we observed TFST and TSST. TFST and TSST reflect the duration of disease and symptom control and incorporate treatment tolerability and patient compliance [22]. Although real-world PFS is used in retrospective studies in oncology, it often requires manual extraction of data from medical charts, potentially slowing research and limiting the number of patients participating in retrospective studies [24]. TFST could be considered a candidate surrogate marker for real-world OS or PFS, although further validation is needed [24–26]. TSST could be considered a proxy for time to second objective disease progression or death, as long as the second subsequent therapy is initiated by disease progression rather than the toxicity of the previous therapy [27]. Our results showed that the median TFST was 6.4 months in both groups of patients, but the ongoing treatment probability was higher in relapsed/progressed patients than in de novo patients from 6.4 months since the initiation of first-line treatment (*P* < 0.001). Median TSST (9.5 vs. 9.9 months, *P* < 0.001) was shorter in de novo patients than in relapsed/progressed patients. Similar results were reported in a study from Japan, although it was conducted in the pre-immunotherapy era and identified patients with metastatic NSCLC treated with chemotherapy. In the study, de novo patients had a worse median PFS from the initiation of first-line chemotherapy than patients with postoperative recurrence (4.2 vs. 5.5 months, *P* < 0.01) [12].

To our knowledge, the mechanism underlying the difference in survival between patients with de novo versus relapsed/progressed NSCLC is unknown. The difference may be attributed to the relatively high tumor burden in de novo patients, as reported in previous studies. For instance, Gibson et al. [10] reported that de novo cohort of NSCLC had more extrapulmonary metastatic sites than relapsed cohort (27% vs. 3%, *P* < 0.001) noting that this could explain the worse prognosis of the de novo cohort. Additionally, Sekine et al. [12] observed that brain and bone metastases were significantly more common in de novo compared to patients with postoperative recurrence, while pulmonary metastases were more frequent in the patients with postoperative recurrence. The relatively limited routine monitoring in de novo patients may contribute to the higher tumor burden in this group. Due to routine monitoring, disease progression is likely to be detected earlier in asymptomatic relapsed/progressed patients, resulting in smaller tumor burden. In contrast, de novo patients are more likely to present with symptoms indicating a more advanced disease stage at the time of detection.

Unlike the differences in OS and TSST, treatment patterns for both de novo and relapsed/progressed patients were similar, except that treatment regimens for relapsed/progressed patients were more varied in first-line therapy. Among patients who received first-line palliative therapy, platinum-based chemotherapy was the most prevalent in both groups, consistent with the results of previous studies [17, 18]. De novo patients used paclitaxel/pemetrexed/gemcitabine + platinum as first-line therapy more frequently than relapsed/progressed patients (92.0% vs. 63.9%). Previous use of the platinum-based regimen as adjuvant therapy may have affected the treatment pattern of the relapsed/progressed group. Although recent studies in the United States reported high use of immunotherapy in the first-line setting [15, 28], a direct comparison is inappropriate because, in our study, immunotherapies were not reimbursable for first-line therapy during the study period, resulting in low use of immunotherapy.

Therapeutic approaches varied in second-line therapy, with no regimen accounting for > 20%. Similarly, previous studies conducted before immunotherapies became prevalent in the United States showed that various chemotherapies were used in advanced NSCLC [16, 29]. In our study, docetaxel was the preferred second-line therapy for both groups of patients. However, previous studies conducted in patients with metastatic NSCLC without *EGFR* or *ALK* mutations and in the post-immunotherapy era showed that immunotherapy was the most common second-line therapy. For example, Simeone et al. [17] reported that nivolumab was the most frequent regimen, accounting for 31% of second-line therapies among patients with metastatic NSCLC, using Flatiron health data from January 2013 to January 2017. Similarly, in a study using Flatiron health data from 2018 to 2019, most patients (50.7%) with metastatic NSCLC used second-line therapy containing immunotherapy [28]. Compared with previous studies, the patients included in this study were treated with chemotherapy more than immunotherapy. The difference might be due to limited patient access to immunotherapy, considering that the study period included the era before immunotherapy reimbursement for the second and subsequent line of therapy, which has been effective since 2017.

This study represents a large-scale, multi-year analysis of prognosis and treatment patterns between patients with de novo and relapsed/progressed NSCLC. In this study, both inpatient and outpatient prescriptions were confirmed as part of the national health insurance system in South Korea based on a fee-for-service delivery system. The results of our study are representative of patients with stage IIIB–IV NSCLC in South Korea, as the database covers nearly the entire Korean population. Considering the impact of the disease stage at the time of the initial diagnosis on OS, our findings highlight the importance of screening for the early detection of NSCLC. In addition, these findings suggest that the stage of the disease at the time of the initial diagnosis should be considered in observational studies and clinical trials as a prognostic factor. To the best of our knowledge, this is the first study to represent the treatment pattern of patients with NSCLC without *EGFR* or *ALK* mutations, divided into de novo and relapsed/progressed patients.

This study had several limitations. First, we could not obtain genomic information as the HIRA database does not provide them. Therefore, *EGFR* mutation and *ALK* translocation statuses were inferred based on the use of *EGFR* and *ALK* tyrosine kinase inhibitors, not by molecular testing. In addition, patients with *EGFR* mutations or *ALK* translocations may not have been treated with targeted therapies. However, *EGFR* and *ALK* tyrosine kinase inhibitors are the most commonly recommended regimens for patients with *EGFR* and *ALK* mutations [30]. Similarly, patients with SCLC were excluded based on first-line treatment, which was used as an alternative for biopsy results. Also, we were unable to identify PD-L1 status in the HIRA database. However, patients receiving immunotherapies may have a certain level of PD-L1 expression, given that PD-L1 expression levels are used to determine reimbursement for immunotherapies in South Korea. Second, it was impossible to identify the actual status of the cancer stage in the HIRA database. However, we used an operational definition from a previous study that identified patients with stage IIIB–IV NSCLC using the HIRA claims data [20], and the approach was discussed with clinical experts. Third, there is a possibility of misclassifying palliative therapy initiated within 6 months of thoracic surgery as adjuvant therapy. This could underestimate the OS observed in the relapsed/progressed group and misclassify second-line therapy as first-line therapy. However, the difference in OS remained significant when we changed the operational definition to 5 months. In addition, the operational definition was based on a previous study and the results of consultation with clinical experts that setting a narrower gap can misclassify adjuvant therapy as first-line palliative therapy [20]. Finally, there could be potential confounders, and some prognostic variables were not available, such as squamous cell histology and smoking history.

## Conclusions

Our study confirmed that patients with de novo NSCLC have worse prognoses than those with relapsed/progressed stage IIIB–IV NSCLC without *EGFR* or *ALK* mutations. De novo patients had poorer OS and TSST after the initiation of palliative therapy compared with relapsed/progressed patients under similar treatments. These findings suggest that the stage of the disease at the time of initial diagnosis should be considered in observational studies and clinical trials as a prognostic factor.

## Electronic supplementary material

Below is the link to the electronic supplementary material.

Supplementary Material 1

## Data Availability

The datasets generated and/or analysed during the current study are not publicly available because the Korean Health Insurance Review and Assessment Service (HIRA) does not allow researchers to provide data personally or share publicly but are available from the corresponding author on reasonable request.

## Abbreviations

ALK: Anaplastic lymphoma kinase
CCI: Charlson comorbidity index
CI: Confidence interval
EGFR: Epidermal growth factor receptor
HIRA: Health Insurance Review and Assessment Service
HR: Hazard ratio
ICD-10: International Classification of Disease-10th revision
NSCLC: Non-small cell lung cancer
OS: Overall survival
PD-L1: Programmed death-ligand 1
PFS: Progression-free survival
SCLC: Small cell lung cancer
SD: Standard deviation
TFST: Time to first subsequent therapy or death
TSST: Time to second subsequent therapy or death

## References

[1] Sung H, Ferlay J, Siegel RL, Laversanne M, Soerjomataram I, Jemal A (2021). Global Cancer Statistics 2020: GLOBOCAN estimates of incidence and Mortality Worldwide for 36 cancers in 185 countries. CA Cancer J Clin.

[2] SEER Cancer Stat Facts. : Lung and Bronchus Cancer. National Cancer Institute. Bethesda, MD, https://seer.cancer.gov/statfacts/html/lungb.html.

[3] American Cancer Society. Cancer Facts & Fig. 2021. Atlanta: American Cancer Society; 2021.

[4] American Cancer Society. Non-small-cell lung cancer survival rates, by stage. (2011–2017). https://www.cancer.org/cancer/non-small-cell-lung-cancer/detection-diagnosis-staging/survival-rates.html.

[5] Chen R, Manochakian R, James L, Azzouqa AG, Shi H, Zhang Y (2020). Emerging therapeutic agents for advanced non-small cell lung cancer. J Hematol Oncol.

[6] Schwartzberg L, Korytowsky B, Penrod JR, Zhang Y, Le TK, Batenchuk C (2019). Real-world clinical impact of Immune checkpoint inhibitors in patients with Advanced/Metastatic Non-Small Cell Lung Cancer after Platinum Chemotherapy. Clin Lung Cancer.

[7] Nokihara H, Kijima T, Yokoyama T, Kagamu H, Suzuki T, Mori M et al. Real-world treatments and clinical outcomes in Advanced NSCLC without actionable mutations after introduction of Immunotherapy in Japan. Cancers (Basel). 2022;14(12). 10.3390/cancers14122846 PMC9220782 35740512

[8] Bozorgmehr F, Kazdal D, Chung I, Kirchner M, Magios N, Kriegsmann M (2021). De Novo Versus secondary metastatic EGFR-Mutated non-small-cell Lung Cancer. Front Oncol.

[9] Yu HA, Sima CS, Hellmann MD, Naidoo J, Busby N, Rodriguez K (2015). Differences in the survival of patients with recurrent versus de novo metastatic KRAS-mutant and EGFR-mutant lung adenocarcinomas. Cancer.

[10] Gibson AJW, Li H, D’Silva A, Tudor RA, Elegbede AA, Otsuka S (2019). Comparison of clinical characteristics and outcomes in relapsed Versus De Novo Metastatic Non-Small Cell Lung Cancer. Am J Clin Oncol.

[11] Moore S, Leung B, Wu J, Ho C (2019). Survival implications of De Novo Versus Recurrent Metastatic Non-Small Cell Lung Cancer. Am J Clin Oncol.

[12] Sekine I, Nokihara H, Yamamoto N, Kunitoh H, Ohe Y, Tamura T (2009). Comparative chemotherapeutic efficacy in non-small cell lung cancer patients with postoperative recurrence and stage IV disease. J Thorac Oncol.

[13] Dawood S, Broglio K, Ensor J, Hortobagyi GN, Giordano SH (2010). Survival differences among women with de novo stage IV and relapsed breast cancer. Ann Oncol.

[14] Lobbezoo DJ, van Kampen RJ, Voogd AC, Dercksen MW, van den Berkmortel F, Smilde TJ (2015). Prognosis of metastatic breast cancer: are there differences between patients with de novo and recurrent metastatic breast cancer?. Br J Cancer.

[15] Nadler E, Arondekar B, Aguilar KM, Zhou J, Chang J, Zhang X (2021). Treatment patterns and clinical outcomes in patients with advanced non-small cell lung cancer initiating first-line treatment in the US community oncology setting: a real-world retrospective observational study. J Cancer Res Clin Oncol.

[16] Davis KL, Goyal RK, Able SL, Brown J, Li L, Kaye JA (2015). Real-world treatment patterns and costs in a US Medicare population with metastatic squamous non-small cell lung cancer. Lung Cancer.

[17] Simeone JC, Nordstrom BL, Patel K, Klein AB (2019). Treatment patterns and overall survival in metastatic non-small-cell lung cancer in a real-world, US setting. Future Oncol.

[18] Abernethy AP, Arunachalam A, Burke T, McKay C, Cao X, Sorg R (2017). Real-world first-line treatment and overall survival in non-small cell lung cancer without known EGFR mutations or ALK rearrangements in US community oncology setting. PLoS ONE.

[19] Kim S, Kim MS, You SH, Jung SY (2020). Conducting and reporting a Clinical Research using korean Healthcare Claims Database. Korean J Fam Med.

[20] Lee JS, Hong JH, Sun S, Won HS, Kim YH, Ahn MS (2019). The impact of systemic treatment on brain metastasis in patients with non-small-cell lung cancer: a retrospective nationwide population-based cohort study. Sci Rep.

[21] Jang SC, Kwon SH, Min S, Jo AR, Lee EK, Nam JH (2022). Optimal Indicator of Death for using Real-World Cancer patients’ data from the Healthcare System. Front Pharmacol.

[22] Campbell BA, Scarisbrick JJ, Kim YH, Wilcox RA, McCormack C, Prince HM. Time to Next Treatment as a meaningful endpoint for trials of primary cutaneous lymphoma. Cancers (Basel). 2020;12(8). 10.3390/cancers12082311 PMC7463470 32824427

[23] Quan H, Li B, Couris CM, Fushimi K, Graham P, Hider P (2011). Updating and validating the Charlson comorbidity index and score for risk adjustment in hospital discharge abstracts using data from 6 countries. Am J Epidemiol.

[24] Walker B, Boyd M, Aguilar K, Davies K, Espirito J, Frytak J (2021). Comparisons of real-world time-to-event end points in Oncology Research. JCO Clin Cancer Inform.

[25] Branchoux S, Sofeu CL, Gaudin AF, Kurt M, Moshyk A, Italiano A (2022). Time to next treatment or death as a candidate surrogate endpoint for overall survival in advanced melanoma patients treated with immune checkpoint inhibitors: an insight from the phase III CheckMate 067 trial. ESMO Open.

[26] Stewart M, Norden AD, Dreyer N, Henk HJ, Abernethy AP, Chrischilles E (2019). An exploratory analysis of real-world end points for assessing outcomes among immunotherapy-treated patients with Advanced Non-Small-Cell Lung Cancer. JCO Clin Cancer Inform.

[27] Matulonis UA, Oza AM, Ho TW, Ledermann JA (2015). Intermediate clinical endpoints: a bridge between progression-free survival and overall survival in ovarian cancer trials. Cancer.

[28] Stenehjem D, Lubinga S, Betts KA, Tang W, Jenkins M, Yuan Y (2021). Treatment patterns in patients with metastatic non-small-cell lung cancer in the era of immunotherapy. Future Oncol.

[29] McKay C, Burke T, Cao X, Abernethy AP, Carbone DP (2016). Treatment patterns for Advanced Non-Small-cell Lung Cancer after Platinum-containing therapy in U.S. Community Oncology Clinical Practice. Clin Lung Cancer.

[30] Hanna N, Johnson D, Temin S, Baker S, Brahmer J, Ellis PM (2017). Systemic therapy for Stage IV Non-Small-Cell Lung Cancer: American Society of Clinical Oncology Clinical Practice Guideline Update. J Clin Oncol.

