# Supplementary material for: Survival differences between patients with de novo and relapsed/progressed advanced non-small cell lung cancer without epidermal growth factor receptor mutations or anaplastic lymphoma kinase rearrangements
Source: BMC Cancer. 2023 May 29;23:482. doi: 10.1186/s12885-023-10950-y (PMC10226257; doi:10.1186/s12885-023-10950-y)
Supplement: Supplementary file 1 — Supplementary Material 1 [file 12885_2023_10950_MOESM1_ESM.docx]

**Supplementary material: contents**

Supplementary S1 Fig. Study design

Supplementary S2 Fig. Patient flowchart

Supplementary S3 Fig. Results of sensitivity analysis on overall survival when cytotoxic chemotherapy used within 5 months is considered adjuvant therapy

Supplementary S4 Fig. Overall survival of de novo and relapsed/progressed advanced non-small cell lung cancer patients from the 1-year landmark

Supplementary S5 Fig. Overall survival in de novo and relapsed/progressed advanced non-small cell lung cancer stratified by immunotherapy use

Supplementary S1 Table. Treatment regimens for inclusion and exclusion

Supplementary S2 Table. Treatment regimens included in the analysis

Supplementary S3 Table. Hazard ratio for time to first subsequent treatment or death

Supplementary S4 Table. Median overall survival in de novo and relapsed/progressed advanced non-small cell lung cancer stratified by immunotherapy use

Supplementary S5 Table. Top five regimens and their time to treatment discontinuation (median, IQR)

**
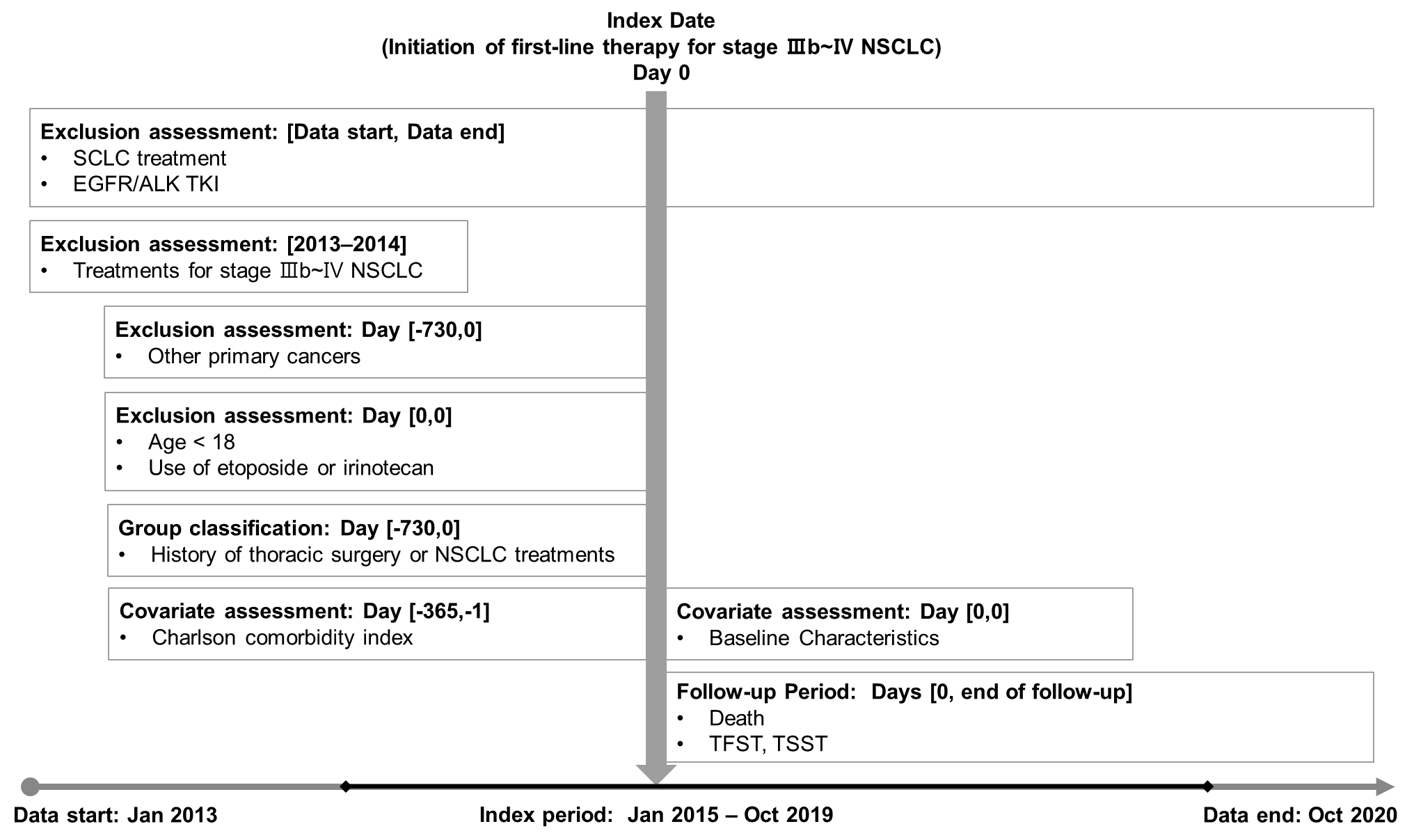
**

**S1 Fig. Study design**

ALK, anaplastic lymphoma kinase; EGFR, epidermal growth factor receptor; NSCLC, non-small cell lung cancer; SCLC, small cell lung cancer; TKI, tyrosine kinase inhibitor

S1 Table. Treatment regimens for inclusion and exclusion

| **Treatment type** | **Incl./excl.** | **Therapy** | **Time frame** |
| --- | --- | --- | --- |
| SCLC treatment | Excluded | belotecan  cyclophosphamide+doxorubicin+cisplatin  cyclophosphamide+doxorubicin+vincristine  cyclophosphamide+etoposide+vincristine  ifosfamide+carboplatin  ifosfamide+doxorubicin  ifosfamide+etoposide+vincristine+carboplatin  topotecan  topotecan+carboplatin  topotecan+cisplatin  topotecan+etoposide  topotecan+ifosfamide | Study period |
| NSCLC first-line palliative treatment  - Chemotherapy | Included | bevacizumab+gemcitabine+cisplatin  bevacizumab+paclitaxel+carboplatin  docetaxel  docetaxel+carboplatin  docetaxel+cisplatin  docetaxel+vinorelbine  gemcitabine  gemcitabine+carboplatin  gemcitabine+cisplatin  gemcitabine+vinorelbine  paclitaxel  paclitaxel+carboplatin  paclitaxel+cisplatin  paclitaxel+ifosfamide  paclitaxel+vinorelbine  pemetrexed+cisplatin  pemetrexed+carboplatin  vinorelbine  vinorelbine+carboplatin  vinorelbine+cisplatin  vinorelbine+ifosfamide  vinorelbine+ifosfamide+cisplatin | Index period |
| NSCLC first-line palliative treatment  - Immunotherapy | Included | atezolizumab (±chemotherapy)  nivolumab (±chemotherapy)  pembrolizumab (±chemotherapy) |  |
| Stage IIIB–IV NSCLC treatment | Excluded | bevacizumab+gemcitabine+cisplatin  bevacizumab+paclitaxel+carboplatin  docetaxel  docetaxel+vinorelbine  docetaxel+vinorelbine+carboplatin  docetaxel+vinorelbine+cisplatin  gemcitabine  gemcitabine+vinorelbine  gemcitabine+vinorelbine+carboplatin  gemcitabine+vinorelbine+cisplatin  irinotecan  paclitaxel+etoposide+carboplatin  paclitaxel+etoposide+cisplatin  paclitaxel+ifosfamide  paclitaxel+ifosfamide+carboplatin  paclitaxel+ifosfamide+cisplatin  paclitaxel+vinorelbine  paclitaxel+vinorelbine+carboplatin  paclitaxel+vinorelbine+cisplatin  pemetrexed  pemetrexed+cisplatin  pemetrexed+carboplatin  atezolizumab  nivolumab  pembrolizumab | 2013–2014 |
| Stage IIIB–IV NSCLC treatment*  - EGFR/ALK TKI | Excluded | afatinib  alectinib  brigatinib  ceritinib  crizotinib  erlotinib  gefitinib  osimertinib | Study period |
| * Regimens reimbursed for adjuvant therapy are not included.  ALK, anaplastic lymphoma kinase; EGFR, epidermal growth factor receptor; NSCLC, non-small cell lung cancer; SCLC, small cell lung cancer; TKI, tyrosine kinase inhibitor | | | |

S2 Table. Treatment regimens included in the analysis

| **Treatment type** | **Treatment Class** | **Therapy** |
| --- | --- | --- |
| First-line  palliative treatment | Chemotherapy | bevacizumab+gemcitabine+cisplatin  bevacizumab+paclitaxel+carboplatin  docetaxel  docetaxel+carboplatin  docetaxel+cisplatin  docetaxel+vinorelbine  gemcitabine  gemcitabine+carboplatin  gemcitabine+cisplatin  gemcitabine+vinorelbine  paclitaxel  paclitaxel+carboplatin  paclitaxel+cisplatin  paclitaxel+ifosfamide  paclitaxel+vinorelbine  pemetrexed+cisplatin  pemetrexed+carboplatin  vinorelbine  vinorelbine+carboplatin  vinorelbine+cisplatin  vinorelbine+ifosfamide  vinorelbine+ifosfamide+cisplatin |
|  | Immunotherapy* | atezolizumab (±chemotherapy)  nivolumab (±chemotherapy)  pembrolizumab (±chemotherapy) |
| Second and subsequent-line palliative treatment | Chemotherapy | docetaxel  docetaxel+carboplatin  docetaxel+cisplatin  docetaxel+vinorelbine  docetaxel+vinorelbine+carboplatin  docetaxel+vinorelbine+cisplatin  etoposide+carboplatin  etoposide+carboplatin+ifosfamide  etoposide+cisplatin  etoposide+cisplatin+ifosfamide  gemcitabine  gemcitabine+carboplatin  gemcitabine+cisplatin  gemcitabine+vinorelbine  gemcitabine+vinorelbine+carboplatin  gemcitabine+vinorelbine+cisplatin  irinotecan  irinotecan+carboplatin  irinotecan+cisplatin  paclitaxel  paclitaxel+carboplatin  paclitaxel+cisplatin  paclitaxel+etoposide+carboplatin  paclitaxel+etoposide+cisplatin  paclitaxel+ifosfamide  paclitaxel+ifosfamide+carboplatin  paclitaxel+ifosfamide+cisplatin  paclitaxel+vinorelbine  paclitaxel+vinorelbine+carboplatin  paclitaxel+vinorelbine+cisplatin  pemetrexed  pemetrexed+cisplatin  pemetrexed+carboplatin  vinorelbine  vinorelbine+carboplatin  vinorelbine+cisplatin  vinorelbine+ifosfamide  vinorelbine+ifosfamide+cisplatin |
|  | Immunotherapy | atezolizumab  nivolumab  pembrolizumab |
| * Immunotherapies are not reimbursed in Korea as the first-line therapy during the study period. Thus, patients treated with immunotherapies in the first line would have paid 100% of the drug cost according to the Health Insurance Review and Assessment Service guidance. | | |

**
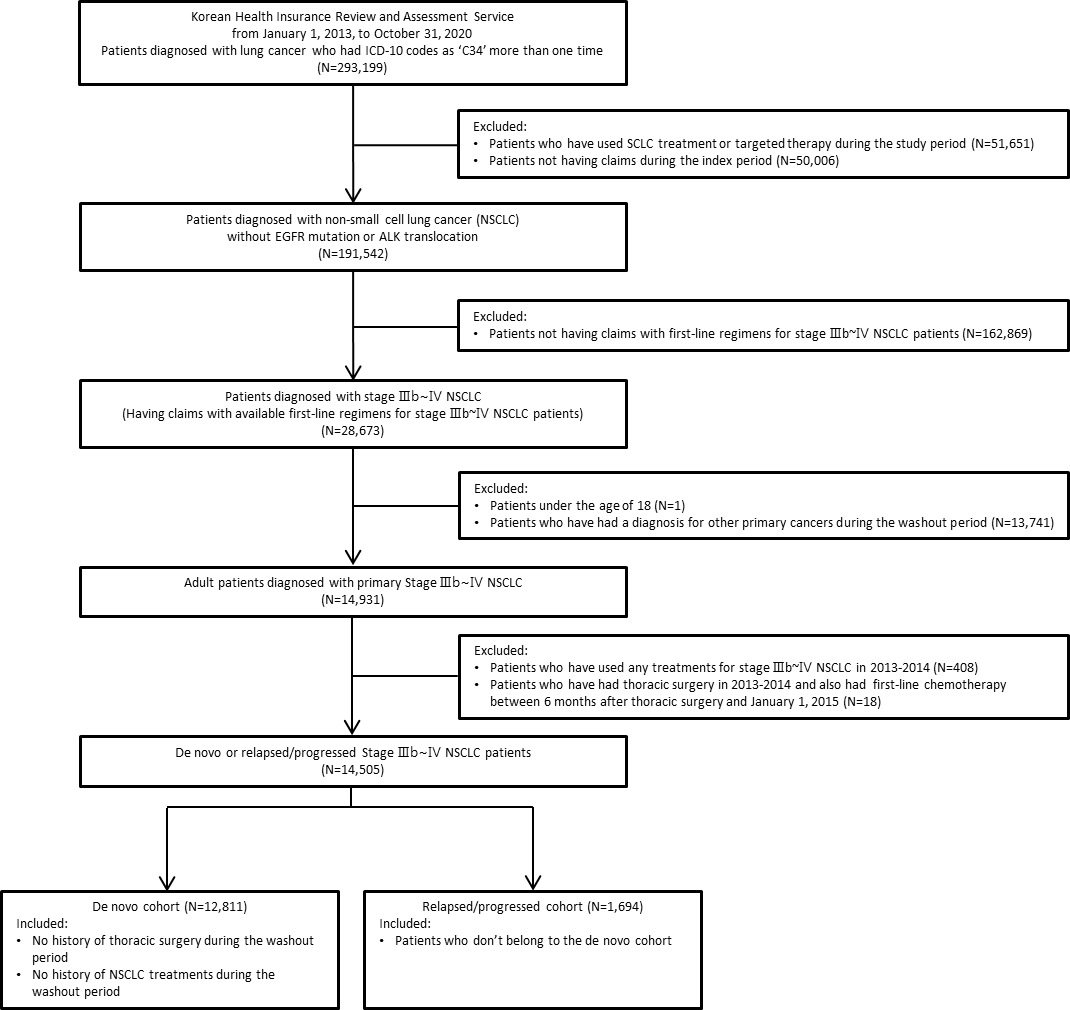
**

**S2 Fig. Patient flow chart**

ALK, anaplastic lymphoma kinase; EGFR, epidermal growth factor receptor; ICD-10, International Classification of disease 10th revision; NSCLC, non-small cell lung cancer; SCLC, small cell lung cancer

**S3 Table. Hazard ratio for time to first subsequent treatment or death**

|  | Hazard ratio (95% CI) |
| --- | --- |

| **Variable** | | **Total (n = 14,505)** | **De novo patients**  **(n = 12,811)** | **Relapsed/progressed patients (n = 1,694)** |
| --- | --- | --- | --- | --- |
| **De novo vs. relapsed/progressed** | | |  |  |
|  | Relapsed/progressed | reference | - | - |
|  | De novo | 1.09 (1.03–1.15) | - | - |
| **Age group** | | |  |  |
|  | < 60 | reference | reference | reference |
|  | 60–69 | 1.08 (1.03–1.14) | 1.10 (1.04–1.16) | 1.00 (0.86–1.16) |
|  | 70–79 | 1.18 (1.12–1.24) | 1.19 (1.13–1.26) | 1.07 (0.92–1.25) |
|  | ≥ 80 | 1.32 (1.22–1.42) | 1.30 (1.20–1.41) | 1.58 (1.19–2.10) |
| **Sex** | | |  |  |
|  | Female | reference | reference | reference |
|  | Male | 1.36 (1.29–1.44) | 1.35 (1.27–1.43) | 1.46 (1.23–1.73) |
| **CCI** | | |  |  |
|  | < 3 | reference | reference | reference |
|  | ≥ 3 | 1.03 (0.99–1.07) | 1.02 (0.98–1.06) | 1.09 (0.97–1.22) |
| **Insurance type** | | |  |  |
|  | National health insurance | reference | reference | reference |
|  | Medical aid or veterans | 1.13 (1.05–1.21) | 1.12 (1.04–1.21) | 1.16 (0.94–1.43) |
| **Type of hospital at initiation of first-line therapy** | | |  |  |
|  | Tertiary hospital | reference | reference | reference |
|  | Others | 1.04 (1.00–1.08) | 1.04 (1.00–1.09) | 1.01 (0.89–1.15) |
| **Geographic region of hospital** | | |  |  |
|  | Capital area | reference | reference | reference |
|  | Metropolitans | 1.01 (0.96–1.06) | 1.03 (0.98–1.08) | 0.88 (0.76–1.03) |
|  | Rural | 0.96 (0.91–1.01) | 0.95 (0.90–1.00) | 1.00 (0.85–1.17) |
| **Index year** | | |  |  |
|  | Pre-immunotherapy | reference | reference | reference |
|  | Post-immunotherapy | 0.79 (0.76–0.82) | 0.79 (0.76–0.82) | 0.81 (0.73–0.91) |
| CCI, charlson comorbidity index; CI, confidence interval | | | | |


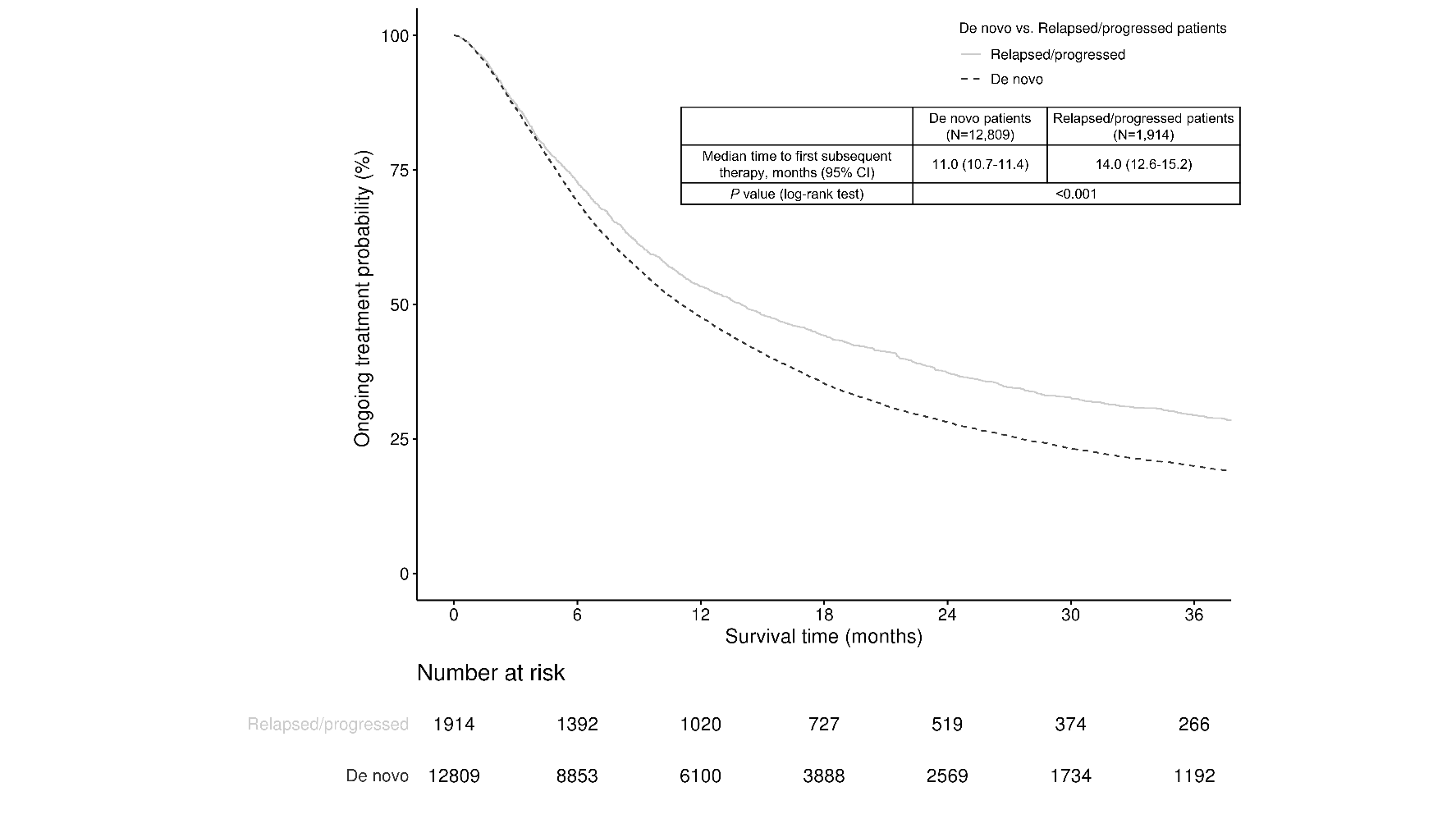


**S3 Fig. Results of sensitivity analysis on overall survival when cytotoxic chemotherapy used within 5 months is considered adjuvant therapy**

CI, confidence interval


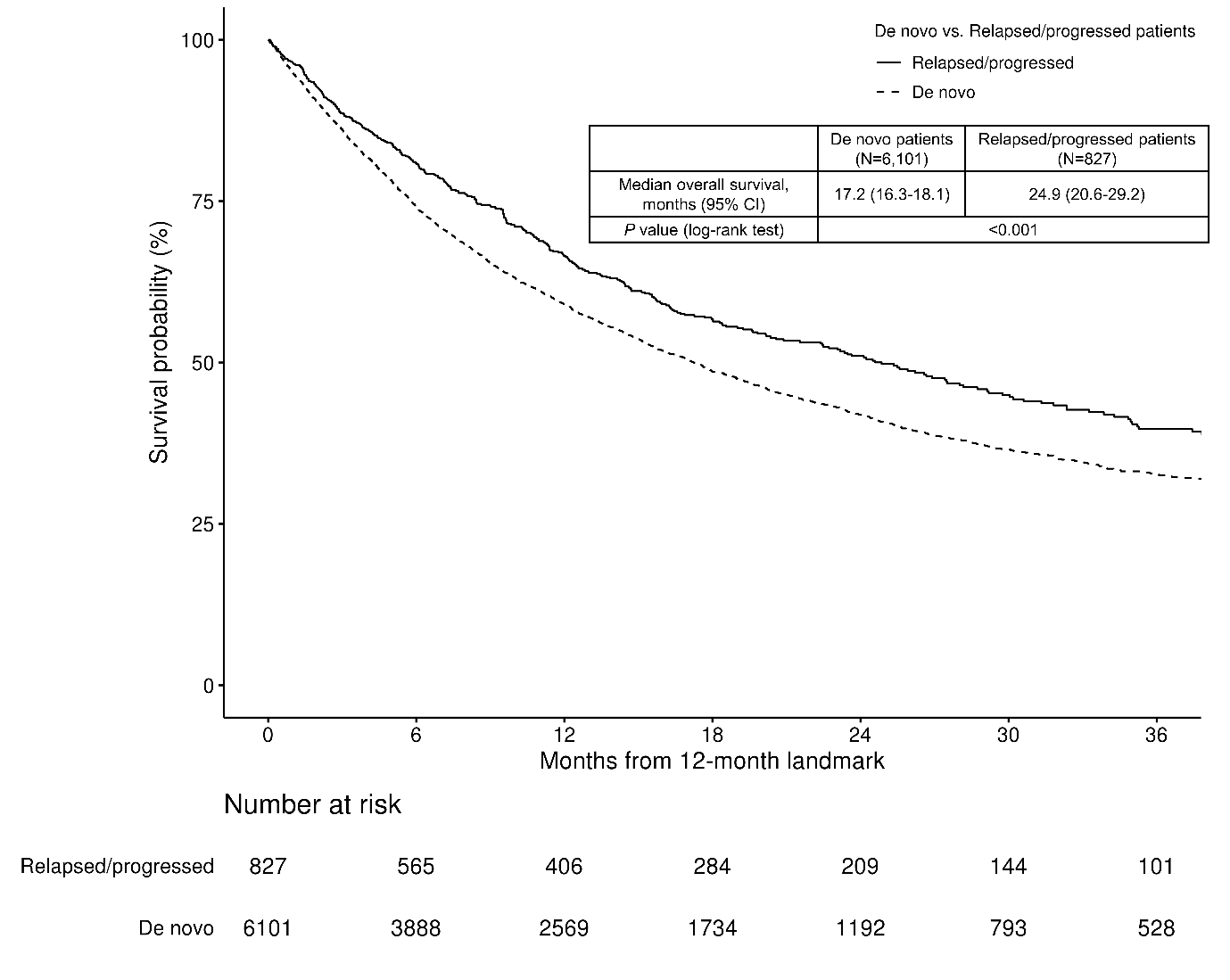


**S4 Fig. Overall survival of de novo and relapsed/progressed advanced non-small cell lung cancer patients from the 1-year landmark**

CI, confidence interval


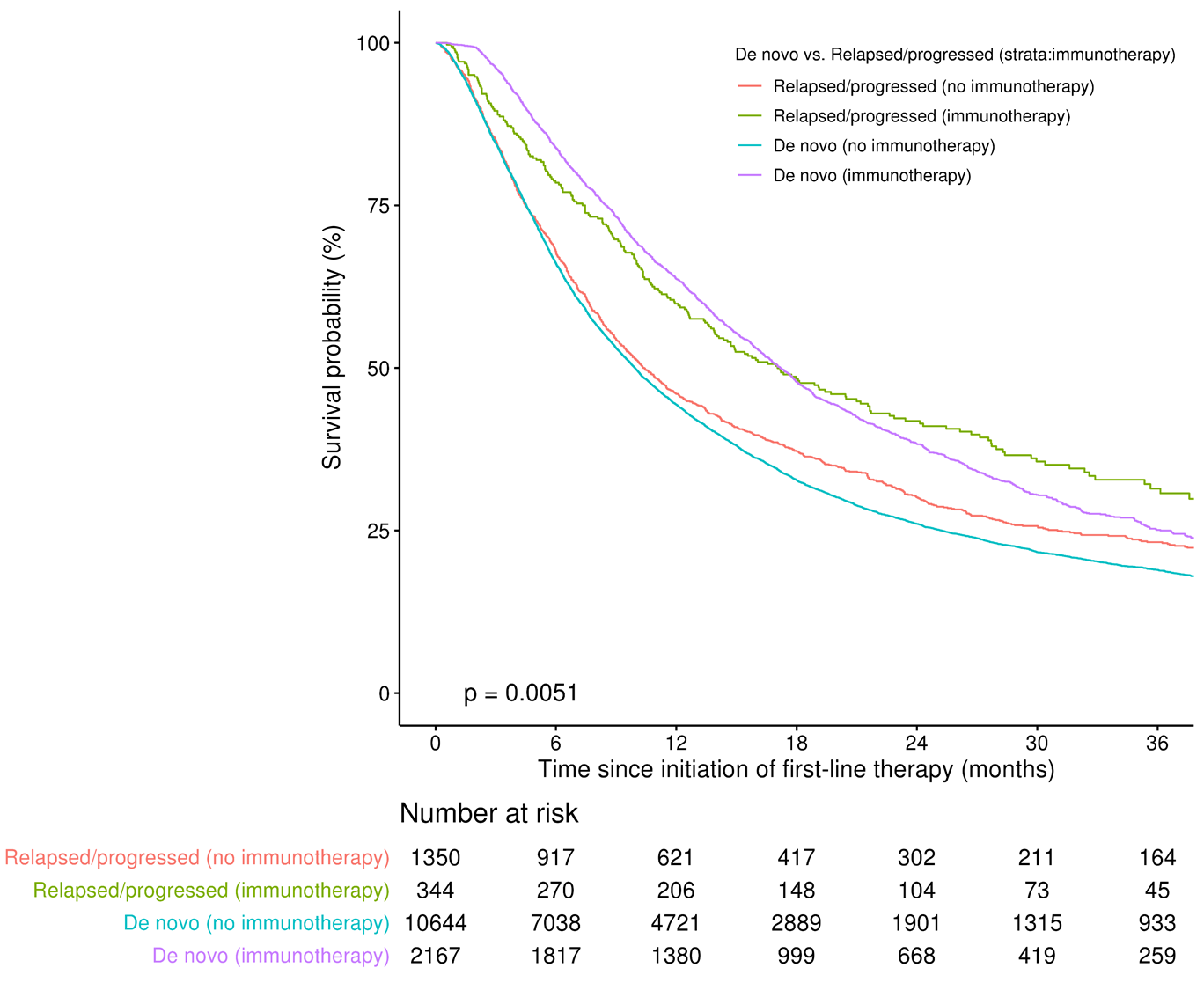


**S5 Fig. Overall survival in de novo and relapsed/progressed advanced non-small cell lung cancer stratified by immunotherapy use**

**S4 Table. Median overall survival in de novo and relapsed/progressed advanced non-small cell lung cancer stratified by immunotherapy use**

|  | Number of patients | Median overall survival (months) | 95% Confidence interval (months) |
| --- | --- | --- | --- |
| De novo (immunotherapy) | 2,167 | 17.2 | 16.4–18.0 |
| De novo (No immunotherapy) | 10,644 | 10.0 | 9.7–10.2 |
| Relapsed/progressed (immunotherapy) | 344 | 17.1 | 14.0–21.6 |
| Relapsed/progressed (No immunotherapy) | 1,350 | 10.4 | 9.5–11.51 |

**S5 Table. Top five regimens and their time to treatment discontinuation (median, IQR)**

|  | Number of patients (%) | | | Time to treatment discontinuation (months), median (IQR) | | |
| --- | --- | --- | --- | --- | --- | --- |
|  | Total | De novo patients | Relapsed/progressed patients | Total | De novo patients | Relapsed/progressed patients |
| First-line regimen | 14,505 (100.0) | 12,811 (100.0) | 1,694 (100.0) | 2.5 (1.6–3.4) | 2.6 (1.7–3.4) | 2.1 (1.4–3.2) |
| Paclitaxel+platinum | 4,758 (32.8) | 4,387 (34.2) | 371 (21.9) | 2.1 (1.8–3.1) | 2.1 (1.8–3.1) | 1.9 (1.5–2.5) |
| Pemetrexed+platinum | 4,063 (28.0) | 3,763 (29.4) | 300 (17.7) | 2.8 (1.8–3.3) | 2.9 (1.8–3.3) | 2.8 (1.7–3.2) |
| Gemcitabine+platinum | 4,042 (27.9) | 3,631 (28.3) | 411 (24.3) | 2.7 (1.4–3.9) | 2.7 (1.4–3.9) | 2.6 (1.3–3.5) |
| Gemcitabine | 430 (3.0) | 337 (2.6) | 93 (5.5) | 1.5 (0.9–3.1) | 1.5 (0.9–3.1) | 1.5 (1.2–3.0) |
| Docetaxel+platinum | 329 (2.3) | 295 (2.3) | 34 (2.0) | 2.8 (1.5–4.3) | 2.9 (1.6–4.4) | 1.5 (0.8–2.8) |
| Others | 883 (6.1) | 398 (3.1) | 485 (28.6) | 2.1 (1.2–3.5) | 2.3 (1.4–3.4) | 1.9 (1.1–3.5) |
| Second-line regimen | 5.973 (100.0) | 5,314 (100.0) | 659 (100.0) | 2.1 (1.3–4.0) | 2.1 (1.4–4.0) | 2.1 (1.3–3.9) |
| Docetaxel | 1,127 (18.9) | 996 (18.7) | 131 (19.9) | 1.7 (1.2–3.0) | 1.7 (1.2–3.0) | 1.7 (1.2–2.9) |
| Gemcitabine+platinum | 946 (15.8) | 860 (16.2) | 86 (13.1) | 2.3 (1.3–3.3) | 2.3 (1.3–3.3) | 2.2 (1.3–3.2) |
| Pembrolizumab | 822 (13.8) | 765 (14.4) | 57 (8.7) | 3.5 (1.5–10.5) | 3.5 (1.5–10.1) | 3.5 (1.6–16.6) |
| Nivolumab | 688 (11.5) | 617 (11.6) | 71 (10.8) | 2.8 (1.4–7.5) | 2.8 (1.4–7.4) | 2.3 (1.3–7.8) |
| Paclitaxel+platinum | 404 (6.8) | 373 (7.0) | 31 (4.7) | 2.0 (1.5–3.0) | 2.0 (1.5–3.0) | 2.2 (1.4–3.2) |
| Others | 1,986 (33.3) | 1,703 (32.1) | 283 (42.9) | 2.0 (1.2–3.6) | 2.0 (1.2–3.6) | 2.0 (1.2–3.5) |
| IQR, interquartile range  All percentages may not add to a total of 100% because of rounding. | | | | | | |
